# Advances in the Treatment of Keratoconus: Epithelial-On (EPI-On) Corneal-Collagen Cross-Linking (CXL) and CXL-Plus Procedures

**DOI:** 10.7759/cureus.51565

**Published:** 2024-01-03

**Authors:** Maaz Khan, Ahmed Ageed

**Affiliations:** 1 Medical Education, Royal Surrey County Hospital, Guildford, GBR; 2 Internal Medicine, University Hospitals of Leicester NHS Trust, Leicester, GBR

**Keywords:** corneal topography, visual acuity measurement, refraction, collagen cross-linking, keratoconus (kc)

## Abstract

Keratoconus (KC) incidence is on the increase. The advent of corneal-collagen cross-linking (CXL) has revolutionized the management of KC. This systematic review looks at the efficacy and complications of two novel treatments within CXL: Epithelial-On (Epi-On) and CXL-plus procedures. Two separate literature searches were carried out up until July 1, 2021. Articles only published in the last two years were included to ensure that only the most recent articles were reviewed. A total of 15 articles were selected for this review. There were varied results regarding the efficacy of Epi-On. No significant difference was found between Epi-On and standard Epithelial-Off (Epi-Off) CXL. However, it was found that Epi-On was inferior to standard CXL in terms of reducing *K*_MAX_. There was a higher risk of progression in patients treated with Epi-On CXL, with an increased rate of patients requiring re-treatment due to the advancement of their KC. While some studies report CXL-plus procedures demonstrate long-term efficacy and safety, a considerable number of studies advise caution, reporting a significant deterioration in corrected distance visual acuity (CDVA). Consequently, a question persists regarding the safest and most efficacious approach, given the lack of robust large randomized controlled trials (RCTs) in the current literature.

## Introduction and background

Keratoconus (KC) is the most common corneal ectasia [1]. With an estimated incidence of one in 2,000 in the general population [2]. Corneal ectasia is a group of eye conditions characterized by the bilateral thinning of the cornea, which can be at the center, paracentral, or periphery.

KC was first described in detail by J. Nottingham in 1854. He comprehensively talked about the epidemiology, presentation, underlying cause, and treatment. However, there were limitations largely due to a lack of technological advances at the time. Today, we know there are several different phenotypes documented, predominately inferior corneal thinning (pellucid marginal degeneration) and generalized corneal thinning (keratoglobus) [3]. It is still uncertain, however, if these are forms of KC or separate entities [3]. In KC, there is bilateral and asymmetrical corneal degeneration that occurs due to corneal thinning, which results in protrusion of the thinner cornea, becoming conical in shape. The conical cornea leads to irregular astigmatism and high myopia. The effect on vision usually becomes apparent in the second decade of life, most commonly during puberty [2].

KC progression is typical until the fourth decade of life when it plateaus [2]. During its progression, it is one of the commonest reasons for keratoplasty in the West, although, with the advent of corneal-collagen cross-linking (CXL), this has reduced [3]. KC affects both genders; studies have failed to identify if any one gender is more at risk. Some studies suggest there is no difference between genders, while others state that males are more at risk; in contrast, some have described females as having a greater prevalence [4]. KC is a global condition affecting people across all backgrounds. There have been studies showing that individuals of certain Asian ethnic origins have up to seven times higher rates of incidence. This has been attributed to consanguineous relationships, which are common, suggesting that there is also a genetic component to KC [5]. Other common associations include eye rubbing, long-term trauma from contact lens use, and allergic eye disease.

Over time, we have been able to understand a lot more about KC. The advent of CXL has revolutionized the management of KC. New protocols are constantly being developed, raising hope for reducing the overall number of patients progressing to the point of requiring surgery. This article review focuses on the efficacy and complications of two novel treatments within CXL: Epi-On CXL and CXL-Plus procedures.

CXL

CXL is a minimally invasive sterile procedure that is mostly performed in an outpatient clinic. It involves the application of riboflavin ultraviolet-A (UVA) and oxygen. This treatment aims to *freeze* the progression of KC via the oxidation pathway. In this photochemical reaction, radicals are created to help create cross-links by interacting with the carbonyl group of collagens. Cross-linking involves the creation of bonds to link one polymer chain to another. During this process, UVA light generates radicals. However, to enhance effectiveness, we use riboflavin as a special photosensitizer in the presence of UVA light. Riboflavin absorbs UVA energy and becomes excited, transitioning into a triplet-excited riboflavin through an exchange mechanism. The presence of oxygen is required to form singlet oxygen (type 2 reaction). Riboflavin, along with a very reactive singlet oxygen (1O_2_), forms an oxygen radical. If oxygen is absent, type one reactions occur. In CXL, both type 1 and type 2 reactions occur [6].

This is the original CXL protocol introduced by Wollensak et al. The first human trials took place in 2003 in Dresden, involving 16 patients with progressive KC. All patients ceased progression after undergoing CXL. Furthermore, 70% had flattening of their steep anterior curvature, and more than 60% had an improvement in their visual acuity. The study reported no serious complications [7]. The protocol included the debridement of the central corneal epithelium following the application of topical anesthesia. Subsequently, a solution of 0.1% riboflavin in 20% dextran was instilled every two minutes for 30 minutes [7].

Transepithelial CXL (Epi-On)

Another modification suggested for the Dresden protocol involves limiting disturbance to the corneal epithelium. This is done to expedite post-procedural healing and protect the endothelium, particularly in individuals with thin corneas. This approach is commonly referred to as *Epi-On* CXL. The advantages of Epi-On CXL include avoiding the risk of postoperative infections, experiencing less intense pain during epithelial healing, and a shorter recovery time. However, a primary challenge with Epi-On CXL is achieving effective diffusion of riboflavin into the corneal stroma. Strategies to enhance penetration include prolonging contact time, altering the permeability of the corneal epithelium, or modifying the properties of the riboflavin itself [8].

CXL-Plus

Visual rehabilitation in KC involves the CXL procedure as well as contact lens therapy to address myopia and astigmatism. However, there is a growing demand for complete visual rehabilitation through surgery, achieved by combining CXL with a refractive procedure. These approaches are termed *CXL-Plus* procedures. One such CXL-Plus procedure is CXL in conjunction with photorefractive keratectomy (PRK). This is termed the *Athens Protocol*, involving same-day, topography-guided partial PRK and CXL [9].

## Review

Materials and methods

This review focused on the recent advancements in treatments of KC. The field of KC treatment is vast, and a plethora of options are available. Therefore, a literature search focusing on two specific treatment options was undertaken. The two treatment options are *transepithelial CXL* (TEL) and *CXL-Plus procedures*. The review aims to explore the efficacy and side effect profile of each treatment option, comparing different variations and determining which is superior.

Two separate literature searches were conducted up to July 1, 2021, using the main databases Ovid MEDLINE and Ovid EMBASE. As mentioned previously, novel treatments in KC are many; therefore, a literature search of only two types of treatments was carried out. Key concepts used in the search included "Keratoconus," "Transepithelial CXL," and "combined CXL and PRK." For a more detailed view of the searches conducted on the respective databases, please refer to Appendices A-D. Mesh and free-text terms were used to capture all the relevant references. 

**Figure 1:**
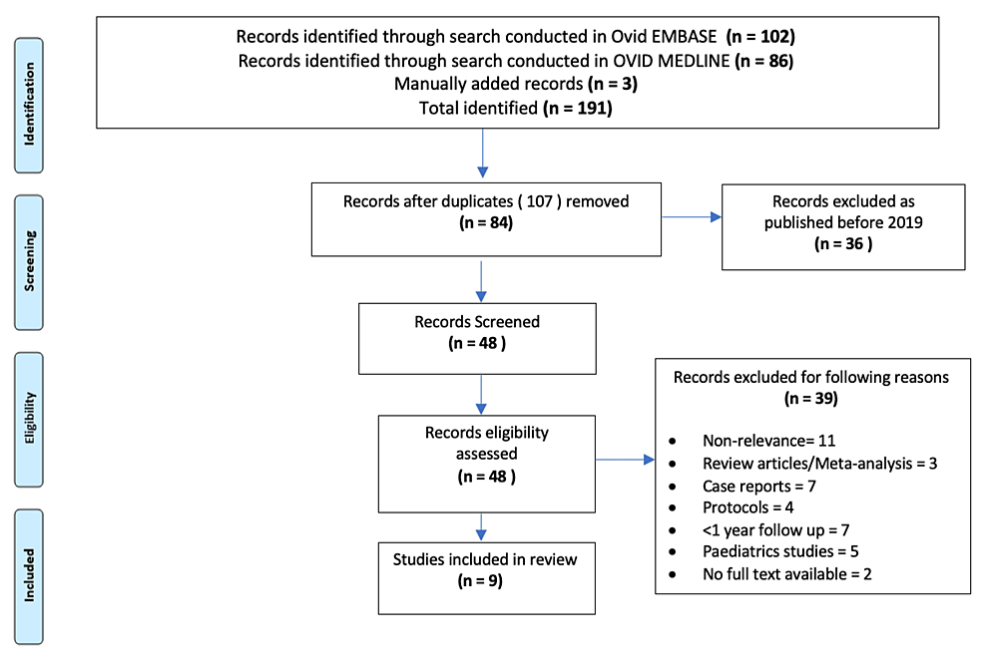
PRISMA flow diagram depicting the Epi-On search process. PRISMA, Preferred Reporting Items for Systematic Reviews and Meta-Analyses; Epi-On, Epithelial-On

First, a search for Epi-On CXL was conducted on the respective databases; refer to Appendices A-B for the search terms. All the references were exported to Endnote, and duplicates were removed, leaving 84 references to be screened. References published within a two-year time frame (2019-2021) were included. This process resulted in 42 references that were reviewed and assessed for eligibility. The inclusion criteria were defined to select only articles in English or those with available translations. Priority was given to randomized controlled trials (RCTs) and studies with large sample sizes. Articles had to meet specific criteria, excluding review papers, conference abstracts, case reports, or protocols. The focus was on studies based on TEL in adult populations, with a minimum follow-up of 12 months. Studies were also excluded if deemed irrelevant, such as those incorporating other forms of KC treatments, for example, intracorneal ring segment (ICRS) protocols. The steps taken are shown in the PRISMA (Preferred Reporting Items for Systematic Reviews and Meta-Analyses) flow diagram (Figure 1) [10]. After the exclusion process, nine studies were selected.

**Figure 2:**
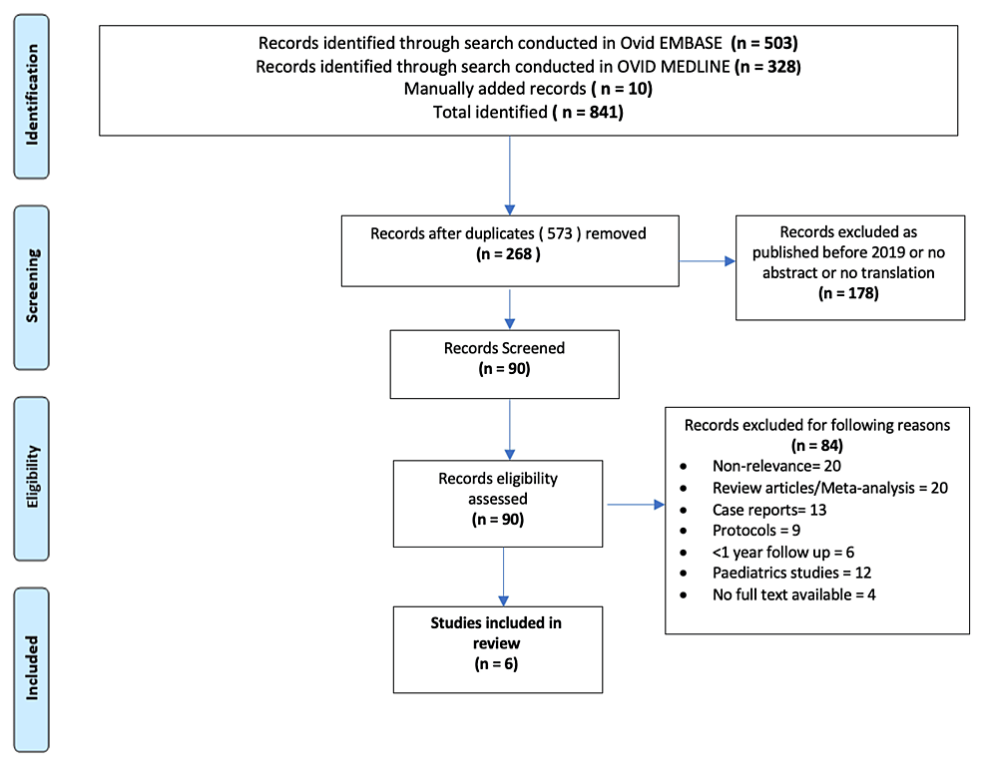
PRISMA flow diagram for the CXL-Plus search. PRISMA, Preferred Reporting Items for Systematic Reviews and Meta-Analyses; Epi-On, Epithelial-On; CXL, corneal-collagen cross-linking

Next, a search for CXL-Plus was conducted on the respective databases; refer to Appendices C-D for the search terms. The methodology applied for the Epi-On CXL (as explained in the last paragraph) was also used for the CXL-Plus search. The steps taken are shown in the PRISMA flow diagram (Figure 2) [10]. After exclusion, six studies were selected.

Results

TEL Epi-On

Efficacy of TEL: Godefrooij et al. carried out a prospective longitudinal cohort study at a tertiary center in the Netherlands. They compared TEL (labeled as TEL CXL in this study) with several different variations of Epi-Off CXL. A total of 670 eyes were monitored, with follow-up extending over one year. The conventional Dresden protocol, labeled as SCXL-MedioCROSS in their study, was used as the reference. The SCXL refers to standard CXL, not accelerated CXL (ACXL); MedioCROSS refers to the riboflavin drops used. The primary outcome was to look at the independent effects of various treatment protocols by evaluating the maximum keratometry and mean keratometry(*K*_Mean_) one year after the treatments. They used multivariable regression to provide the *β* coefficients (*β*) for *K*_MAX_ (maximum anterior sagittal curvature) and *K*_Mean_. Godefrooij et al. reported that when multivariable linear regression analysis was performed compared to the reference group (SCXL-MedioCROSS), the TEL CXL group (*β* = 1.422; *P *= 0.001) performed significantly worse than the reference group, along with the Epi-Off ACXL-Vibex Rapid group, the SCXL-Meran group, and ACXL-Collagex [11]. All the aforementioned treatment modalities were less effective in preventing KC progression as they ended up increasing the *K*_MAX _value. Regarding *K*_Mean_, the same four treatment modalities also exhibited significant differences compared to the reference group, all resulting in an increase in *K*_Mean_.

When looking at the secondary outcomes of Godefrooij et al., the corrected distance visual acuity (CDVA) in all treatment protocols was either unchanged or improved. Godefrooij et al. concluded that TEL CXL while being time-consuming also had a less favorable outcome regarding halting the progressive nature of KC. The most effective protocols are the Epi-Off CXL [11]. Of all eyes in the TEL CXL, 33.3% required re-treatment due to post-CXL progression of KC. These patients underwent an additional epi-off CXL.

Lombardo et al. [12] carried out an RCT of TEL CXL against standard Epi-Off CXL to prevent the progression of KC, with *K*_MAX_ being the primary outcome. This trial was different from other studies as TEL was carried out via the noninvasive technique called iontophoresis. The RCT randomized 22 eyes for TEL and 12 eyes for standard CXL, with clinical outcomes of *K*_MAX_, visual acuity (UDVA and CDVA), and corneal aberrations evaluated via ophthalmic examinations throughout a two-year follow-up period. The examination included Placido-disk corneal topography and anterior OCT for *K*_MAX_. All the procedures were performed by a single surgeon at a single-site study. At baseline, the mean *K*_MAX_ was similar between the two groups: TEL (54.7 ± 4.0 Diopters [D]) and standard CXL (54.7 ± 4.3 D) (*P* = 0.87). At the end of 24 months, there was an improvement in *K*_MAX_ in both groups, with means of 53.7 ± 3.7 D (*P* = 0.07) and 53.2 ± 4.9 D (*P* < 0.001) in the TEL CXL and the standard CXL groups, respectively. More than a -0.5 improvement in *K*_MAX_ was observed in 50% of eyes in the TEL group and 83% in the standard CXL group. However, in the TEL group, three patients experienced a *K*_MAX_ steepening of more than 0.5 D, with two of the three cases showing an increase of 1.00 D, indicating progression of KC during the same period. In the control group, no cases of *K*_MAX_ increased at the 24-month stage. The two cases that demonstrated *K*_MAX_ progression of more than 1.00 D were among the youngest at enrollment (<21 years of age). Overall, when examining *K*_MAX_ values at the 24-month stage and comparing them to baseline measurements, there was no statistically significant difference between the two treatment groups (*P* = 0.06) [12].

When looking at visual acuity, Lombardo et al. reported that the mean uncorrected distance visual acuity (UDVA) improved in both treatment groups. The mean UDVA significantly improved in the TEL group from 0.80 ± 0.19 Logarithm of the Minimum Angle of Resolution (logMAR) at baseline to 0.48 ± 0.36 logMAR at 24 months (*P* < 0.001). Meanwhile, in the control group, it improved from 0.65 ± 0.30 logMAR to 0.32 ± 0.29 logMAR (*P* = 0.01). At the end of the two-year follow-up period, no statistically significant difference in UDVA improvement between the groups was found (*P* = 0.78). Interestingly, in the TEL group, the two cases where patients experienced a steepening in their *K*_MAX_ showed significant visual improvements when their visual outcomes were evaluated. Patient A400 exhibited a clinically significant visual improvement of -0.3 logMAR, while patient A220 showed a change of -0.1 logMAR at the 24-month interval [12].

Yuksel et al. reported that Epi-On CXL performed significantly worse compared to Epi-Off CXL in halting the progression of KC [13]. Yuksel et al. conducted a study comparing accelerated Epi-On CXL to accelerated Epi-Off CXL in 42 eyes with KC. *K*_MAX_, *K*_mean_, and BCVA (best corrected visual acuity), among other clinical outcomes, were measured with a total 30-month follow-up period. *K*_MAX_ (maximum anterior sagittal curvature) was seen as an indicator of the stability of KC after the CXL procedure. Progression, defined as an increase of more than 1 D in the *K*_MAX_ value, often occurs with a rising *K*_MAX_ (in diopters). Both treatment groups, after the 30-month follow-up period, succeeded in preventing the progression of KC. The difference in the preoperative *K*_MAX_ values between the two groups was statistically nonsignificant. In the Epi-Off CXL group, the preoperative *K*_MAX_ was 55.2 ± 3.6 D, and at 18 months post-CXL, it was 53.8 ± 4.1 D. The Epi-On preoperative *K*_MAX _value was 58.0 ± 4.9 D, and at 18 months, it was 56.9 ± 3.9 D. The reduction in *K*_MAX_ at the 18-month stage between the two groups was significantly more reduced in the Epi-Off CXL when compared to TEL CXL (*P *= 0.04). Therefore, this indicates that when evaluating the *K*_MAX_ values at the 18-month interval, Epi-Off CXL was more effective at halting the progression of KC. Regarding BCVA with spectacles in logMAR, Yuksel et al. reported no significant differences in the preoperative values in either group (*P *= 0.25). Overall, the BCVA significantly increased by approximately 1 Snellen line in both Epi-Off and Epi-On CXL at the end of the follow-up period (24 and 30 months), with no statistical difference between the two groups [13].

Cifariello et al. reported a significant difference in BCVA when comparing Epi-On and Epi-Off CXL. They conducted a two-year follow-up comparative study comparing Epi-Off versus Epi-On (transepithelial) CXL [14]. The study included 40 eyes from 32 patients in Italy. Twenty eyes were randomly assigned to CXL-Epi-Off (group 1) and 20 to the TEL group 2 (TEL Epi-On), with a follow-up of two years. Each patient group was operated by the same surgeon. All patients underwent complete ophthalmological testing. At the end of the two-year follow-up period, key findings were significant increases in BCVA to baseline in both groups (*P *= 0.01). In contrast to Yuksel et al., group 2 (Epi-On) exhibited a significantly greater improvement in BCVA compared to the Epi-Off group during the follow-up period. There was an improvement of 0.14 logMAR in group 2 compared to 0.05 in group 1; a statistically significant difference was seen between the BCVA in each group (*P *= 0.01) [14].

**Figure 3:**
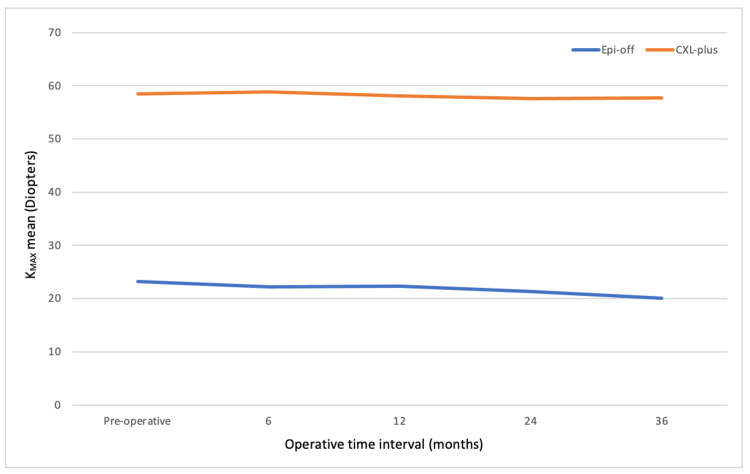
Graph showing the KMAX values at preoperative, six months, 12 months, 24 months, and 36 months.

Arance-Gil et al. carried out a long-term non-randomized prospective study looking at progressive KC [15]. Thirty-one eyes were treated by Epi-Off CXL and 33 were treated by TEL. There was a 36-month follow-up period with variables such as CDVA and *K*_MAX_, among others. The groups were not homogeneous in terms of age, which is an important factor that should be considered. As a result, it was a covariate in the statistical methods. The results showed age did not influence the variables that were studied. Furthermore, it was noted that the Epi-Off CXL compromised more advanced patients with KC. Regarding *K*_MAX,_ both the Epi-On and TEL *K*_MAX_ values remained stable, thereby halting further progression of KC. The preoperative *K*_MAX_ value for the Epi-Off group was 58.49 ± 1.52, and at 36 months, it was 57.68 ± 1.62. The TEL preoperative *K*_MAX_ values were 53.33 ± 1.82, and at 36 months, they were 53.93 ± 1.94. Figure 3 shows the significant changes in *K*_MAX_ values depending on the treatment group.

However, it must be noted that the Epi-Off CXL patient group comprised more advanced patients with KC. At 36 months, the *K*_MAX _values for the TEL group did rise, although very minimally, from 53.33 to 53.93. Arance-Gil et al. also investigated improvements in CDVA following the CXL procedures. Depending on the location of the cone, KC was determined to be either central or paracentral. Significant improvements were seen in CDVA in both groups. Central KC CDVA improved with a mean of -0.2 logMAR, and paracentral KC CDVA increased with a mean of -0.07 logMAR. Patients with central KC experienced a significantly greater increase. Specifically, 13 patients with central KC underwent Epi-Off CXL, compared to six who chose TEL. Conversely, among patients with paracentral KC, 27 opted for TEL, while 18 chose Epi-Off CXL [15].

Complications of TEL

Cifariello et al. reported very few side effects in their study [14]. In the Epi-On CXL group, the researchers reported two cases, with one patient exhibiting Vogt’s striae and another having type 2 corneal haze, out of a total of 20 patients. There was only one patient with reported side effects in the EPI-Off group (Vogt striae). Cifarellio et al. assessed the level of discomfort between Epi-On and Epi-Off through a disease index, specifically the Ocular Surface Disease Index (OSDI), both before treatment and one month later in all patients. There was a statistically significant increase in the OSDI score in the Epi-Off group one month after treatment (*P *= 0.01). At baseline (pretreatment), the OSDI score was 4.85 ± 1.18 and 4.98 ± 1.32 in groups 1 and 2, respectively. One month later, the OSDI scores increased to 13.56 ± 2.15 and 11.26 ± 2.12, respectively [10]. The difference in OSDI scores between the two groups was also statistically significant. Schirmer's and tear break time tests were conducted; however, there were no observed lacrimal issues in either group.

Cifariello et al. reported that the Epi-On group also experienced less pain and photophobia when compared to the Epi-Off group. The Epi-Off group experienced stromal haze (four eyes had posterior stroma haze) although this disappeared after four months.

Yuksel et al., however, reported results contrary to most expectations [13]. This was also a comparative study looking at accelerated Epi-On or Epi-Off CXL in patients with KC. They reported significantly more pain on day 1 in the Epi-On CXL group when compared to the Epi-Off group. The mean score pain score on day 1 of the procedures was 3.0 ± 0.57 in Epi-Off CXL and 3.7 ± 0.95 in Epi-On CXL eyes (*P* = 0.004) [13].

CXL-Plus (Combined CXL and PRK)

Regarding the efficacy of CXL-Plus, Nattis et al. carried out the Topolink study [16]. This was a single surgeon (Rosenberg D) operating on 62 KC eyes underdoing Epi-On CXL and then topography-guided PRK. PRK was carried out using an excimer laser once the eye was stable post-CXL. Stabilization was defined as no change in refraction or topography for at least three months after the CXL procedure. Refractive treatment could only be addressed if treatment of the refractive error would leave a minimum calculated residual stromal bed thickness of at least 300 microns. Therefore, the patients were divided into two subgroups: the refractive group, comprising those treated for topographic irregularities and refractive error, and the nonrefractive group, which had only topographic irregularities treated due to insufficient stromal bed thickness.

When comparing the two subgroups, those patients who underwent the refractive treatment tended to be younger at the time of CXL (34.62 ± 4.52 vs. 42.29 ± 4.81 years; *P* = 0.02). When looking at the time elapsed between CXL and PRK, there was no significant difference between the two groups (*P *= 0.198). The mean time elapsed between CXL and PRK was 30.5 ± 27.99 months. Overall, there was a statistically significant improvement in DVA (three-line improvement: 20/100 to 20/60, *t*-test, *P* = 0.007) as well as CDVA (two-line improvement: 20/50 to 20/30, *t*-test, *P* < 0.001) at the one-year follow-up interval across both groups. Concerning keratometry indices, there was a significant reduction in *K*_MAX_ across the groups (decreased by 3.61 D, *t*-test, *P* = 0.004), as well as a decrease in high-order aberrations (by 3.76 microns, *t*-test, *P* = 0.004).

This study expressed manifest and keratometric refractive errors as power vector B, using power vector analysis. When examining the subgroups in the study, for the nonrefractive group, power vector B at baseline was 6.36 D, and it improved to 5.18 D post-CXL, indicating an improvement of 1.04 D. At the 12-month post-PRK, however, power vector B had worsened to 6.835 D and remained higher than the baseline. In the refractive group, power vector B at baseline was 6.588 D, and by the end of the 12-month post-interval, it had considerably improved to 3.748 D. When comparing baseline B values with any timepoint interval in the nonrefractive group, it was statistically insignificant. In the refractive group, however, when comparing baseline B values with time intervals, significant improvement was found between baseline and six-month post-PRK (*P* = 0.02) and 12-month post-PRK (*P* = 0.04) [16].

Iqbal et al. carried out a prospective comparative study in patients with KC who had documented progression [17]. The study incorporated 125 eyes that were operated upon and completed a 24-month follow-up period. The study compared the results between standard Epi-Off Dresden CXL (group A) to the CXL-Plus procedure of accelerated Epi-Off (AXL) CXL along with immediate same-session standard non-topography PRK (group B). Regarding the keratometry (k) readings, both groups showed a reduction in the postoperative (postop) period. The major difference in the two groups regarding k readings was time. In group A, the improvement in k readings was slow and progressive throughout the follow-up period, whereas in group B, the improvement was immediate and then plateaued throughout the follow-up period. In group A, the *K*_MAX_ values were 48.64 ± 1.34, 48.57 ± 1.28, and 46.61± 1.11 (*P *= 0.003) at the baseline, first month, and 24th month periods, respectively. In group B, the *K*_MAX_ values were 49.30 ± 0.49, 46.37 ± 0.68, and 47.07 ± 0.56 at the preoperative, first month, and 24th month follow-up periods, respectively. Refractive outcomes were also analyzed in both groups. Group A demonstrated a highly significant reduction in the myopic component of 2.04 D postoperatively at the 24-month stage (*P *< 0.001). This improvement was attributed to the clinically significant sphere reduction (1.97 ± 0.62 D at the 24-month interval) achieved by the Dresden protocol (standard CXL) in group A. In group A, there was an insignificant postop cylindrical reduction (*P *< 0.1). Meanwhile, in group B, there were also significant reductions in the myopic components. Regarding myopic improvements, this improved from 2.89 ± 1.05 to 1.38 ± 0.57 D (*P *<0.05). The postop cylindrical reduction in group B was significant at 1.65 ± 0.55 D (*P* < 0.001), unlike group A [17].

Similar to Iqbal et al., Rattan et al. [18] also explored simultaneous PRK alongside CXL in patients with KC. Rattan et al. carried out a retrospective nonrandomized interventional study of non-topography-guided PRK and standard Dresden protocol of CXL. Thirty-four patients were evaluated at one, three, six, and 12 months postoperatively. Clinical measurements included uncorrected visual acuity (UCVA), CDVA, and k readings, with visual acuity values converted to logMAR notation. Using student paired t-test the results showed a statistically significant improvement in the UCVA means, with stable readings at the one-year postop period (P = 0.0001). Four eyes (11.74%) had no change in their UCVA measurements. In contrast, 53.6% of eyes achieved 20/20 or better UCVA, a level not observed in any eye at baseline. Additionally, 83.9% exhibited UCVA better than 20/40 at 12 months, as opposed to 12.9% at baseline. PRK refers to the goal of correcting up to 70% of the astigmatism and some of the spherical components. Rattan et al. study showed that at the 12-month stage, there was a significant reduction in the manifest cylindrical component (-2.05 ± 1.27 D at baseline to -0.35 ± 0.16 D at 12 months). Regarding the k readings, the results showed that *K*_MAX _improved initially postop and then continued to improve throughout the follow-up period (49.3 ± 0.52 D at baseline to 48.8 ± 0.63 D at three months and 47.4 ± 0.44 D at 12 months). The paired t-test showed a significant difference (*P* = 0.0001) in the average keratometry and keratometry max reading at the preoperative and 12-month stages. Overall, the UCVA, postop refractive outcomes, and keratometry outcomes showed significant improvements at the three-month visit with stabilization and continued improvement till the 12-month follow-up visit [18].

Tamayo et al. [19] conducted a pilot study examining at wavefront-guided PRK along with simultaneous ACXL in patients with KC. High-order aberrations do play an important role in reduced visual acuity. Therefore, Tamayo et al. hypothesized that correcting these aberrations through wavefront-guided PRK might be a superior alternative to topography-guided PRK. Forty-seven eyes were included in this prospective interventional case series, with 20 eyes lost to follow-up at the six- and 12-month postop outcomes for UDVA, CDVA, and steep keratometry. At the 12-month stage, there was a significant improvement in both mean UDVA (0.77 ± 0.35 vs. 0.08 ± 0.12) and mean CDVA (0.10 ± 0.09 vs. 0.02 ± 0.04 logMAR) observed (*P* <= 0.01). There was no reported loss of any lines in CDVA, while 40.7% of eyes had gained either one or more lines and 18.5% had gained two or more lines of CDVA. The keratometry readings showed that baseline steep *K*s had significantly reduced from 45.94 ± 2.1 to 42.65 ± 2.78 D (*P* < 0.01).

Kanellopoulos, the founder of the Athens Protocol, reported the 10-year outcomes of the CXL-Plus procedure known as the Athens Protocol [9]. A total of 144 eyes were followed up for a mean period of 128 ± 4 months. Kanellopoulos confirmed long-term efficacy for patients with KC after the follow-up period with minimal change from outcomes measured at the one-year interval. Overall, 94.4% demonstrated stabilization of their corneal ectasia, with maximum keratometry (*K*_MAX_) significantly decreasing from 50.57 ± 2.8 to 46.17 ± 1.18 D at one year and 44.75 ± 2.14 D at 10 years (*P* < 0.01). Looking at refractive outcomes, 3.5% showed overcorrection (hyperopic shift). The mean UDVA improved from baseline to 10 years (0.19 ± 0.17 to 0.55 ± 0.19). CDVA also improved from 0.59 ± 0.21 at baseline to 0.81 ± 0.19 at 10 years [9].

Complications of CXL-Plus

This section focuses on the complications reported from studies that looked at CXL-Plus procedures. Moraes et al. [20] carried out a retrospective nonrandomized interventional study examining 26 eyes with KC that underwent PRK followed by standard CXL in the same session. Focusing on the complications, postop CDVA had significantly decreased. Thirteen eyes, constituting 50% of the total, experienced a loss of at least one line, with seven eyes (27%) losing two or more lines. Moraes et al. documented corneal haze at each postop follow-up and graded it on a scale from 0 to 4. Corneal haze grade 1 was observed in 15 (57.7%) eyes, grade 2 in six (23.1%) eyes, and grade 3 in one (3.8%) eye [20]. There was no haze in four (15.4%) eyes. Using Spearman’s correlation, there was a significant correlation between the postop corneal haze seen and the CDVA loss (*P *= 0.001). The onset of clinically significant haze that contributed to CDVA loss was identified at three months postop in two eyes (same patient), six months postop in four eyes (two patients), and one year postop in seven eyes (five patients). The haze was intrastromal in all eyes, as is typically observed after CXL. In some cases, however, the haze was intense. The eye with grade 3 corneal haze developed a persistent deep corneal scar accompanied by severe irregular astigmatism. As a result, three lines of CDVA were lost, and the patient ultimately required deep anterior lamellar keratoplasty (DALK) surgery [20].

Iqbal et al. also examined complications in their study, where group A underwent standard CXL, and group B involved eyes undergoing combined PRK and ACXL. The significant findings included one (1.3%) eye in group B that had an irreversible stromal scar leading to a permanent corneal opacity. Two eyes, one in each group had a persistent epithelial defect, which completely healed and had no effect on the postop visual acuity. All the other complications were resolved within the expected period. Common complications included pain and photophobia in both groups (63.8% and 76.1%, respectively). Corneal haze was present in seven (12.1%) eyes in group A as opposed to four (5.9%) eyes in group B, which disappeared within the first six months in group A and the first month in group B [17].

Discussion

The incidence of KC has increased two to three times in the last 10 to 15 years, as reported in some studies. However, other studies have indicated even higher increases, ranging from five to 10 times [21]. The difference in number backs up the fact that KC is a disease that is still not completely understood. The etiology and pathogenesis, as explained in the earlier sections of this study, are something that we are continuing to learn and understand. The field of KC, however, has been transformed with the advent of CXL. Since the advent of CXL, new protocols have been formulated constantly from the original CXL protocol. Protocols formed include those such as TEL and CXL-Plus procedures, which have been further discussed in the Results section. This section aims to delve into the importance and relevance of the results around TEL CXL and CXL-Plus efficacy and complications.

TEL Epi-On CXL

TEL (Epi-On) CXL has frequently been of limited utility in patients with KC because maintaining the corneal epithelium has been believed to diminish the absorption of riboflavin, thereby potentially reducing the effectiveness of riboflavin and UVA in the CXL process. In this literature search, studies have reported TEL as being statistically similar when compared to standard Epi-Off CXL. When comparing TEL to standard Epi-Off CXL, Arance-Gil et al. reported that the *K*_MAX _ values in both groups were stable, indicating that KC progression had been halted [15]. However, it must be noted that the Epi-Off CXL patient group comprised more advanced patients with KC. Despite having more advanced cases, the *K*_MAX_ in the Epi-Off reduced more than the TEL group, thereby suggesting that for the more advanced cases, Epi-Off CXL seems to be a superior alternative to TEL. Arance-Gil et al. also examined the location of the cone in KC, whether it was central or paracentral. It was reported that there was a higher improvement in CDVA in those with central KC. This is thought to be due to the central area's proximity to the radiation during the CXL procedure. Being central exposes it to a higher intensity of radiation compared to the paracentral region. The central CDVA improvement has also been reported in other studies when examining the location of the cone in patients with KC [22]. This means that patients with a central cone location undergoing CXL may experience better outcomes. 

In their comparison of TEL against various formulations of riboflavin in Epi-Off CXL, Godefrooij et al. reported their primary outcome as assessing *K*_MAX_ one year after treatment [11]. Using multivariable linear regression, their results showed that TEL performed significantly worse than the standard EPI-Off CXL. TEL CXL resulted in the worsening of *K*_MAX_, which means that TEL CXL failed to halt the progression of KC. Looking into the results in more detail, one in three eyes in the TEL CXL group required re-treatment due to KC progression. Therefore, findings reported by Godefrooij et al. suggest that TEL is inferior to the standard Epi-Off CXL. Overall, it fails to halt the progression of KC, and one in three patients will require re-treatment. These results are further backed up by other studies that also report KC progression and instability post-TEL CXL when compared to standard CXL [14,23]. When it comes to the riboflavin formulation, Goodefrooij et al. report Meran riboflavin as the only solution that was associated with progression. Regarding UDVA and CDVA, there were no significant differences between any of the treatment groups; the values either improved or remained the same. It has been widely reported that these parameters are usually similar, and no significant differences are found, which is consistent with previous reports [23].

Yuksel et al., in conjunction with Godefrooij et al., also reported that TEL CXL performed less favorably when compared to Epi-Off CXL [13]. There was a significant difference in the *K*_MAX_ values at the end of the follow-up between the two groups; although TEL did prevented progression, it fared significantly worse than the Epi-Off CXL. When looking at BCVA, at the end of the follow-up (24 and 30 months), there was no significant difference between the two groups. To summarize, Yuksel et al. reported that both Epi-Off and TEL halted KC, but Epi-Off was significantly better than TEL. Looking at the visual outcomes regarding BCVA, there was no difference in the two groups as found in previous studies.

Iontophoresis is another approach that can be utilized when undertaking TEL CXL. Lombardo et al. provided high-quality evidence regarding the efficacy of TEL [12]. Lombardo et al. carried out an RCT comparing TEL with standard Epi-Off CXL over two years. Across both groups, there was an overall improvement in *K*_MAX_, with no statistical difference between the two. The results were interesting as three patients in the TEL group had progression (two patients increased more than 1 D), whereas none in the standard CXL. Lombardo et al. reported no significant difference in UDVA between the two groups at the end of the two-year follow-up. However, looking at the two cases that had a significant progression of their KC, they ended up having a clinically significant improvement in their UDVA. This poses the question of whether KMAX is a suitable clinical marker on which we should base our findings so heavily. As demonstrated in this study, the change in *K*_MAX_ does not always follow a strict pattern and correlates with the changes seen in visual acuity. This is consistent with other similar studies that have questioned *K*_MAX_ as it has failed to correlate with visual acuity outcomes [24].

Complications of TEL seem to be very limited in the studies found in this literature search. The main advantage that TEL offers to patients over standard Epi-Off CXL is the reduced pain experienced, less risk of infection, and faster visual recovery as the epithelium is not debrided. Cifariello et al. reported there was a significant difference in discomfort one month after treatment between the TEL and Epi-Off groups, with the TEL discomfort index being significantly less than the Epi-Off [14].

Contrary to most studies reported, Yuksel et al. found that there was significantly more pain on day 1 in the TEL group than in the Epi-Off group. One thing to consider is that Yuksel et al. did an accelerated form of TEL that was compared to an accelerated Epi-Off CXL technique. This was a more time-efficient method; however, it incorporated higher increasing UVA fluency. It should be noted that Yuksel et al. reported findings regarding the modified accelerated Epi-On CXL technique and not the standard Epi-On technique [15]. 

CXL-Plus

The objective of the CXL-Plus procedure, compared to standard CXL, is to provide not only the stabilization of KC but also to address refractive issues, including CDVA, and high-order aberrations (HOAs). The issue arising with CXL-Plus procedures is that by undertaking significant refractive procedures on unstable KC, you may not achieve favorable stable outcomes and undesirable complications. Sequential (a second procedure done at a later date) has the added complication of increased haze formation due to the activation of fibroblasts post-PRK [25]. The 10-year outcomes of a CXL-Plus procedure, known as the Athens Protocol, demonstrate that same-day CXL-Plus procedures are safe and exhibit long-term efficacy. Of the 144 eyes, 94.4% showed a significant reduction and stabilization of KC over the 10-year period, accompanied by improvements in UDVA and CDVA.

Iqbal et al. compared AXL CXL-Plus to standard Epi-Off CXL in a comparative study. The study found that AXL CXL-Plus is a quicker way to reduce astigmatic and myopic errors of KC with stability over a long time [13]. In contrast, the standard Epi-Off CXL has a slower yet powerful flattening effect of the anterior cornea, with a surprising significant reduction also in the myopic component; this, however, is much slower than CXL-Plus. Both groups had significant reductions in their *K*_MAX_. However, when comparing the results, the standard CXL had a greater reduction in *K*_MAX _with a better long-term profile. This result tells us that standard Epi-Off CXL is more effective at flattening the anterior corneal surface than AXL in the long run. Due to the long-term effects of standard Epi-Off and the myopic and astigmatic reduction from AXL CXL-Plus, the authors suggested that the best combination was standard Epi-Off CXL along with PRK for the best results. 

We can observe the outcomes of Iqbal et al.'s recommendations for combining standard Epi-Off and CXL-Plus by referring to the study conducted by Rattan et al. This study involved simultaneous PRK alongside CXL in 34 patients with KC. The study results indicated a significant difference in both *K*_MAX_ and uncorrected visual acuity (UCVA), reflecting post-refractive outcomes from baseline to the 12-month follow-up visit [17]. There was a reduction in *K*_MAX_ by 1.9 D at the end of the follow-upalong with a reduction of the cylindrical component by 1.7 D. When comparing these results with those of Iqbal et al., the reduction in *K*_MAX_ was not as pronounced as expected, but there was a more significant reduction in the cylindrical component. This implies that standard Epi-Off CXL with PRK performs similarly to ACXL with PRK regarding *K*_MAX_, although the former does offer long-term benefits in the stabilization of KC. This study argues that by doing same-day PRK and CXL, you can avoid the undesired outcomes of haze formation, as it reported no haze or adverse outcomes. However, it should be noted that the calculated statistical power of this study was below 1 and, therefore, must be looked at with caution. 

Epi-On CXL and CXL-Plus represent additional modifications to CXL procedures. Nattis et al. showed in their TOPOLINK study using power vector analysis that those patients who underwent refractive treatment had more favorable manifest and keratometric outcomes compared to patients who did not [16]. The TOPOLINK study involved all patients undergoing Epi-On CXL, followed by either refractive treatment or addressing topographic irregularities, depending on stromal bed thickness. The refractive treatment group patients at the end of the 12-month follow-up had a significant improvement from baseline in their *K*_MAX_, corneal astigmatism, UDVA, and CDVA. These remained stable throughout the follow-up, unlike the other group. However, interestingly, the total HOAs after treatment did not reduce significantly from baseline. From the nonrefractive group, there was a hypothesis that just treating topographic irregularities would improve CDVA. This was not the case, and no other significant difference was found in any parameters measured. *K*_MAX_ in the nonrefractive group did not exhibit any significant difference, unlike the refractive group, despite both groups undergoing CXL procedures. This raises the question of how undergoing the refractive treatment contributes to a higher reduction in *K*_MAX_. Other studies have consistently reported similar results, indicating that undergoing refractive treatment leads to a greater reduction in *K*_MAX_ [26,27].

Nattis et al. failed to see a significant improvement in HOAs after their study. Tamayo et al., however, their study incorporated the use of wavefront-guided PRK alongside ACXL in patients with KC to target these HOAs. In theory, correcting HOAs and low-order aberrations improves retinal image and contrast, subsequently leading to better visual refractive outcomes in patients with KC [28]. At the end of their study, Tamayo et al. reported insignificant improvements in HOA, including mean coma, spherical aberration, and total root mean square (RMS). These improvements, however, did lead to significant improvements in mean UDVA, mean CDVA, and keratometric readings [19]. Findings from Tamayo et al. suggest that wavefront-guided interventions have the potential to target HOAs. However, they also noted that in advanced patients with KC, topography-guided PRK may be the superior option. This approach of utilizing topography-guided PRK in advanced cases was also supported by Kanellopoulos et al. They argued that in highly abnormal KC corneas that were beyond the limits of a wavefront-measuring device, topography-guided PRK is more efficient [9].

Complications related to CXL-Plus procedures are a topic that is studied in detail. Moraes et al. conducted a study examining 26 eyes that underwent simultaneous PRK and CXL. Their significant results raise questions and advise caution regarding the well-established CXL-Plus procedure [20]. Moraes et al. reported 50% of eyes losing at least one line, with clinically significant haze reported in all but four eyes. Using Spearman’s correlation, they concluded that there was a significant correlation between the postop haze and loss of CDVA lines. One patient with grade 3 haze lost three lines of CDVA and ended up requiring DALK surgery. This finding is notable, as corneal haze has not been reported as an issue in any of the other studies, except for Iqbal et al., who documented a single patient experiencing irreversible stromal scarring following PRK-CXL. However, overall Iqbal et al. reported that an overwhelming majority of the corneal haze was insignificant and disappeared [17]. Examining the study by Moraes et al., there were unaddressed limitations in the research. The CXL-Plus procedure did not adhere to established CXL-PRK procedures, primarily because the ablation depth used was higher than the standard, potentially inducing different effects on the cornea. Furthermore, they failed to mention whether CDVA assessments, when conducted, were performed with rigid contact lenses or glasses for the most accurate measurements. Regarding corneal haze, steroids were not prescribed beyond the first month post-op. This limitation on their part suggests that they missed the opportunity to assess the efficacy of prolonged steroid use in combating corneal haze. Guell et al., in a study similar to Moraes et al., reported an improvement in stromal haze when patients were prescribed steroids for an extended duration [29].

Limitations

TEL and CXL-Plus procedures are relatively new, resulting in limited research available on them. The literature review searches were conducted on two scientific databases, potentially overlooking research available on other databases. Strict criteria were applied to the searches to maintain manageable article numbers, although this did result in limiting the selected numbers.

An important constraint on this literature review is the scarcity of gold-standard RCTs concerning TEL and CXL-Plus. This suggests that the research quality is not currently sufficient to significantly influence clinical practice. Conducting future RCTs on TEL and CXL-Plus procedures is imperative. Furthermore, comparing the research is exceedingly difficult due to the limited number of studies. As several studies employ different treatment plans or versions of TEL or CXL-Plus, drawing firm conclusions from various publications can be challenging.

## Conclusions

When discussing TEL as a procedure within the context of CXL, we can conclude that it is a safe intervention, as no serious adverse effects have been reported in any of the studies reviewed. The primary advantage of TEL is that a majority of studies indicate it to be a less painful procedure compared to Epi-Off CXL. However, additional studies are required to ascertain whether modified accelerated TEL is equally as less painful as standard TEL. As for the efficacy of TEL, it can be concluded that the majority of studies indicate TEL performs less favorably when compared to Epi-Off CXL. A consistent finding across all studies is that patients undergoing TEL CXL have a higher likelihood of progression, making them more likely to necessitate re-treatment when compared to Epi-Off CXL. Furthermore, we observe a trend indicating significant differences in *K*_MAX_ values, yet there appears to be no difference in final refractive outcomes, such as UDVA and CDVA. Therefore, we must question whether *K*_MAX_ is the most clinically appropriate marker to use when assessing KC.

CXL-Plus procedures are new novel treatment procedures that are being increasingly studied. These procedures offer both CXL and refractive procedures. However, RCTs are needed to take place, which include larger cohorts of patients with an increased follow-up period. These studies must investigate the safety and efficacy of CXL-Plus procedures. Current studies tell us that CXL-Plus procedures are efficacious in halting KC progression and improving refractive outcomes, generally with a favorable side effect profile. Significant side effects include corneal haze, which, as noted by Moraes et al., can result in the loss of lines of CDVA as a complication. However, questions remain about whether sequential or same-day is the best approach in CXL-Plus procedures. The advantages of CXL-Plus include the integration of refractive procedures with CXL, providing enhanced stabilization of KC and improved refractive outcomes. Finally, a limitation identified in the literature search was the scarcity of RCTs with large patient cohorts. Moving forward, it is crucial to include more robustly reported RCTs examining TEL and the CXL-Plus procedure to establish high-level evidence-based recommendations.

## Appendices

Appendix A 

**Table 1:** Ovid EMBASE 1946-July 2021 -* (Epi-On). Epi-On, Epithelial-On; CXL, corneal-collagen cross-linking

# ▲	Searches	Results
2	(keratoconus or corneal ectasia).mp. [mp=title, abstract, heading word, drug trade name, original title, device manufacturer, drug manufacturer, device trade name, keyword, floating subheading word, candidate term word]	10205
3	1 or 2	10205
4	(transepithelial corneal collagen cross linking or TEL CXL or transepithelial CXL or Epithelium on corneal collagen crosslinking or Epithelium on CXL or Epi on corneal collagen cross linking or Epi on CXL).mp. [mp=title, abstract, heading word, drug trade name, original title, device manufacturer, drug manufacturer, device trade name, keyword, floating subheading word, candidate term word]	119
5	3 and 4	102

Appendix B

**Table 2:** Ovid MEDLINE 1946 to July 2021 -* (Epi-On). Epi-On, Epithelial-On; CXL, corneal-collagen cross-linking

# ▲	Searches	Results
2	(keratoconus or corneal ectasia).mp. [mp=title, abstract, original title, name of substance word, subject heading word, floating sub-heading word, keyword heading word, organism supplementary concept word, protocol supplementary concept word, rare disease supplementary concept word, unique identifier, synonyms]	8227
3	1 or 2	8227
4	(transepithelial corneal collagen cross linking or TEL XL or transepithelial CXL or Epithelium on corneal collagen crosslinking or Epithelium on XL or Epi on corneal collagen cross linking or Epi on XL).mp. [mp=title, abstract, original title, name of substance word, subject heading word, floating sub-heading word, keyword heading word, organism supplementary concept word, protocol supplementary concept word, rare disease supplementary concept word, unique identifier, synonyms]	97
5	3 and 4	86

Appendix C 

**Table 3:** Ovid EMBASE 1946-July 2021 - (CXL-Plus). 2019 onwards. CXL, corneal-collagen cross-linking

# ▲	Searches	Results
1	exp keratoconus/	9217
2	(keratoconus or corneal ectasia). mp. [mp=title, abstract, heading word, drug trade name, original title, device manufacturer, drug manufacturer, device trade name, keyword, floating subheading word, candidate term word]	10205
3	1 or 2	10205
4	exp photorefractive keratectomy/	3042
5	(Combined CXL Photorefractive Keratectomy or Combined corneal collagen cross linking Photorefractive Keratectomy or PRK or Athens Protocol).mp. [mp=title, abstract, heading word, drug trade name, original title, device manufacturer, drug manufacturer, device trade name, keyword, floating subheading word, candidate term word]	3056
6	4 or 5	4702
7	3 and 6	503

Appendix D 

**Table 4:** Ovid MEDLINE 1946 to July 2021 - (CXL-Plus). CXL, corneal-collagen cross-linking

# ▲	Searches	Results
1	exp keratoconus/	5739
2	(keratoconus or corneal ectasia).mp. [mp=title, abstract, original title, name of substance word, subject heading word, floating sub-heading word, keyword heading word, organism supplementary concept word, protocol supplementary concept word, rare disease supplementary concept word, unique identifier, synonyms]	8227
3	1 or 2	8227
4	exp photorefractive keratectomy/	3367
5	(Combined CXL Photorefractive Keratectomy or Combined corneal collagen cross linking Photorefractive Keratectomy or PRK or Athens Protocol).mp. [mp=title, abstract, original title, name of substance word, subject heading word, floating sub-heading word, keyword heading word, organism supplementary concept word, protocol supplementary concept word, rare disease supplementary concept word, rare disease supplementary concept word, unique identifier, synonyms]	2670
6	4 or 5	4293
7	3 and 6	328

## References

[1] Gordon-Shaag A, Millodot M, Shneor E, Liu Y (2015). The genetic and environmental factors for keratoconus. Biomed Res Int.

[2] Rabinowitz YS (1998). Keratoconus. Surv Ophthalmol.

[3] Davidson AE, Hayes S, Hardcastle AJ, Tuft SJ (2014). The pathogenesis of keratoconus. Eye (Lond).

[4] Krachmer JH, Feder RS, Belin MW (1984). Keratoconus and related noninflammatory corneal thinning disorders. Surv Ophthalmol.

[5] Georgiou T, Funnell CL, Cassels-Brown A, O'Conor R (2004). Influence of ethnic origin on the incidence of keratoconus and associated atopic disease in Asians and white patients. Eye (Lond).

[6] Raiskup F, Spoerl E (2013). Corneal crosslinking with riboflavin and ultraviolet A. I. principles. Ocul Surf.

[7] Wollensak G, Spoerl E, Seiler T (2003). Riboflavin/ultraviolet-a-induced collagen crosslinking for the treatment of keratoconus. Am J Ophthalmol.

[8] Sorkin N, Varssano D (2014). Corneal collagen crosslinking: a systematic review. Ophthalmologica.

[9] Kanellopoulos AJ, Asimellis G (2014). Keratoconus management: long-term stability of topography-guided normalization combined with high-fluence CXL stabilization (the Athens Protocol). J Refract Surg.

[10] Page MJ, McKenzie JE, Bossuyt PM (2021). The PRISMA 2020 statement: an updated guideline for reporting systematic reviews. BMJ.

[11] Godefrooij DA, Roohé SL, Soeters N, Wisse RP (2020). The independent effect of various cross-linking treatment modalities on treatment effectiveness in keratoconus. Cornea.

[12] Lombardo M, Serrao S, Lombardo G, Schiano-Lomoriello D (2019). Two-year outcomes of a randomized controlled trial of transepithelial corneal crosslinking with iontophoresis for keratoconus. J Cataract Refract Surg.

[13] Yuksel E, Cubuk MO, Yalcin NG (2020). Accelerated epithelium-on or accelerated epithelium-off corneal collagen cross-linking: Contralateral comparison study. Taiwan J Ophthalmol.

[14] Cifariello F, Minicucci M, Di Renzo F (2018). EPI-off versus Epi-on corneal collagen cross-linking in keratoconus patients: a comparative study through 2-year follow-up. J Ophthalmol.

[15] Arance-Gil Á, Villa-Collar C, Pérez-Sanchez B, Carracedo G, Gutiérrez-Ortega R (2021). Epithelium-Off vs. transepithelial corneal collagen crosslinking in progressive keratoconus: 3 years of follow-up. J Optom.

[16] Nattis AS, Rosenberg ED, Donnenfeld ED (2020). One-year visual and astigmatic outcomes of keratoconus patients following sequential crosslinking and topography-guided surface ablation: the TOPOLINK study. J Cataract Refract Surg.

[17] Iqbal M, Elmassry A, Tawfik A (2019). Standard cross-linking versus photorefractive keratectomy combined with accelerated cross-linking for keratoconus management: a comparative study. Acta Ophthalmol.

[18] Rattan SA, Alshamarti S, Mutashar MK, Al-Salem KM (2019). Non-topography-guided photorefractive keratectomy combined with accelerated collagen cross linking for treatment of keratoconus. Int J Ophthalmol.

[19] Tamayo GE, Castell C, Vargas P, Polania E, Tamayo J (2020). High-resolution wavefront-guided photorefractive keratectomy and accelerated corneal crosslinking for stabilization and visual rehabilitation of keratoconus eyes. Clin Ophthalmol.

[20] Moraes RL, Ghanem RC, Ghanem VC, Santhiago MR (2019). Haze and visual acuity loss after sequential photorefractive keratectomy and corneal cross-linking for keratoconus. J Refract Surg.

[21] Godefrooij DA, de Wit GA, Uiterwaal CS, Imhof SM, Wisse RP (2017). Age-specific incidence and prevalence of keratoconus: a nationwide registration study. Am J Ophthalmol.

[22] Bottós KM, Schor P, Dreyfuss JL, Nader HB, Chamon W (2011). Effect of corneal epithelium on ultraviolet-A and riboflavin absorption. Arq Bras Oftalmol.

[23] Zhang X, Zhao J, Li M, Tian M, Shen Y, Zhou X (2018). Conventional and transepithelial corneal cross-linking for patients with keratoconus. PLoS One.

[24] Li W, Wang B (2017). Efficacy and safety of transepithelial corneal collagen crosslinking surgery versus standard corneal collagen crosslinking surgery for keratoconus: a meta-analysis of randomized controlled trials. BMC Ophthalmol.

[25] Shalchi Z, Wang X, Nanavaty MA (2015). Safety and efficacy of epithelium removal and transepithelial corneal collagen crosslinking for keratoconus. Eye (Lond).

[26] Padmanabhan P, Radhakrishnan A, Venkataraman AP, Gupta N, Srinivasan B (2014). Corneal changes following collagen cross linking and simultaneous topography guided photoablation with collagen cross linking for keratoconus. Indian J Ophthalmol.

[27] Krueger RR, Kanellopoulos AJ (2010). Stability of simultaneous topography-guided photorefractive keratectomy and riboflavin/UVA cross-linking for progressive keratoconus: case reports. J Refract Surg.

[28] Wigledowska-Promienska D, Zawojska I (2007). Changes in higher order aberrations after wavefront-guided PRK for correction of low to moderate myopia and myopic astigmatism: two-year follow-up. Eur J Ophthalmol.

[29] Güell JL, Verdaguer P, Elies D, Gris O, Manero F (2014). Late onset of a persistent, deep stromal scarring after PRK and corneal cross-linking in a patient with forme fruste keratoconus. J Refract Surg.

